# Factors Associated with Public Health Nurses’ Anxiety About Accepting Evacuees During Nuclear Disasters: A Cross-Sectional Study in Oita Prefecture, Japan

**DOI:** 10.3390/healthcare13010045

**Published:** 2024-12-30

**Authors:** Hiro Tsuchiya, Takumi Yamaguchi, Yuko Matsunari

**Affiliations:** 1 School of Health Sciences, Kagoshima University, Kagoshima 890-0075, Japan; k1276806@kadai.jp (H.T.); matsuy@health.nop.kagoshima-u.ac.jp (Y.M.); 2 Research Administration Center, Saitama Medical University, Saitama 350-0495, Japan; 3 Nuclear Safety Research Association, Tokyo 105-0004, Japan; 4 School of Nursing, Tokyo Medical University, Tokyo 160-0022, Japan

**Keywords:** public health nurses, nuclear disaster preparedness, radiation knowledge, evacuee management

## Abstract

**Background/Objectives:** The Fukushima Daiichi Nuclear Power Station accident underscored the critical role of public health nurses (PHNs) in managing evacuees during nuclear emergencies. Despite their importance, PHNs often lack sufficient knowledge and experience, which may make them anxious about this role. This study aimed to investigate the factors associated with PHNs’ anxiety about accepting evacuees and identify strategies to alleviate this anxiety. **Methods:** A cross-sectional survey was conducted among 100 PHNs working in Oita Prefecture, Japan, a region designated to receive evacuees in the event of a nuclear disaster. Data were collected via an online questionnaire assessing radiation knowledge, workplace characteristics, and anxiety about accepting evacuees. Univariate and multivariate logistic regression analyses were performed to identify the predictors of anxiety. **Results:** PHNs working in prefectural offices were 3.4 times more likely to feel anxious about accepting evacuees than those in municipal offices (OR = 3.488, 95% CI = 1.236–10.963, *p* = 0.023). Awareness of evacuation site responsibilities significantly reduced anxiety (OR = 0.412, 95% CI = 0.134–1.211, *p* = 0.110). Overall, knowledge of radiation was low, with only 8% correctly identifying the differences between stochastic and deterministic effects. Online training was the preferred format for education. **Conclusions:** Workplace characteristics and knowledge of evacuation responsibilities significantly influence PHNs’ anxiety levels. Addressing knowledge gaps through tailored, accessible training programs, particularly in online formats, is essential. Strengthening preparedness among PHNs could enhance their confidence and ability to manage evacuees effectively during nuclear disasters.

## 1. Introduction

The Great East Japan Earthquake and the subsequent tsunami on 11 March 2011 triggered the Fukushima Daiichi Nuclear Power Station accident, one of the most severe nuclear disasters in history. The accident released significant amounts of radioactive materials into the environment, causing widespread contamination and raising serious public health concerns [1]. Following this accident, public anxiety and concern about radiation and nuclear disasters have markedly increased. Despite efforts by central and/or local government and research institutions to disseminate accurate information about radiation, many individuals still lack accurate knowledge of the health effects of radiation and continue to harbor vague anxieties and misconceptions [1,2]. Optimizing radiation protection in affected areas requires cooperation among organizations, experts, and affected residents, contributing to the cultivation of a radiation protection culture [3].

PHNs, who manage the health of local residents and interact with them on a daily basis, play a crucial role in disseminating accurate knowledge about the health effects of radiation and radiation protection [4,5,6]. During nuclear disasters, PHNs undertake diverse roles, including coordinating large-scale evacuations among municipalities, providing long-term support, establishing support systems, managing shelters and evacuation guidance, ensuring safety, and offering physical and psychological care [7,8]. Studies have reported that PHNs recognize the need for knowledge and training about radiation and nuclear disasters but often feel their own knowledge is insufficient [5]. Surveys on radiation anxiety and knowledge among PHNs have been conducted in Fukushima Prefecture and other regions hosting nuclear power plants [9]. In Fukushima, the spread of radioactive materials beyond designated evacuation zones led to widespread confusion, highlighting the challenges faced by PHNs in managing unforeseen situations.

Although previous research has provided insights into the anxiety and preparedness of PHNs in areas directly affected by nuclear disasters [9], far less attention has been paid to PHNs in regions designated to receive evacuees. Such recipient areas, while not hosting nuclear facilities themselves, must be prepared to support incoming populations potentially exposed to radiation in the event of a nuclear accident. After the Fukushima accident, the importance of having these recipient regions adequately prepared became evident, especially as they may encounter similar challenges without the direct experience or infrastructure associated with nuclear-hosting regions. Oita Prefecture, for instance, is designated to receive evacuees from the Ikata Nuclear Power Plant in Ehime Prefecture, and all its municipalities are prepared to do so [10]. This means that PHNs in Oita may be required to support residents potentially exposed to radioactive contamination, a responsibility that can induce anxiety due to perceived knowledge gaps.

Therefore, this study aimed to clarify the specific factors associated with PHNs’ anxiety about accepting evacuees during a nuclear disaster in Oita Prefecture, a designated recipient area for evacuees from the Ikata Nuclear Power Plant. By examining how workplace characteristics, radiation-related knowledge, and awareness of evacuation responsibilities influence this anxiety, we sought to identify practical support measures and targeted educational strategies that could alleviate PHNs’ concerns. As this was an exploratory study, these findings will guide the development of future training programs and policy interventions to strengthen PHNs’ preparedness and confidence in managing evacuees during nuclear emergencies.

## 2. Materials and Methods

### 2.1. Study Design

This study used a cross-sectional observational design to explore the factors associated with PHNs’ anxiety about accepting evacuees during a nuclear disaster.

### 2.2. Setting and Participants

This study was conducted in Oita Prefecture, Japan, which is designated as an area to receive evacuees in the event of an emergency at the Ikata Nuclear Power Plant in Ehime Prefecture. Oita Prefecture was selected because, unlike Fukushima or areas hosting nuclear power plants directly, it serves a critical yet understudied role as a designated recipient of evacuees. Understanding the anxiety and preparedness of PHNs in such a receiving region provides insights into broader issues of the nuclear disaster response outside the immediate vicinity of nuclear facilities. The target population included PHNs working at 15 public health institutions within Oita Prefecture, including prefectural health offices and municipal health centers. The data collection period was from February to April 2024.

### 2.3. Data Collection

An explanatory document detailing the study was sent to the heads of the target institutions, and those who agreed to participate returned a document specifying the current number of PHNs at their institution. Subsequently, a URL and QR code for an online questionnaire created using Google Forms were provided to the administrative heads of each participating institution, who then passed this information along to all the PHNs under their supervision. Although the questionnaire link and code were disseminated through institutional channels, explicit instructions made it clear that participation was entirely voluntary and that individual PHNs could choose not to respond without any repercussions. The questionnaire was completed privately and anonymously by each PHN, ensuring that no identifiable personal or institutional information could be traced back to individual respondents. This approach minimized any potential hierarchical pressure and ensured that participants could freely provide their honest opinions.

### 2.4. Measures

The questionnaire was developed for this study, drawing on previously published research related to radiation knowledge and disaster preparedness, as well as the specific context of Oita Prefecture’s role as a designated evacuation site. It did not employ a standardized or validated scale. Rather, it consisted of multiple sections, each with independently developed items intended to explore participants’ knowledge, perceptions, and anxiety regarding nuclear disaster scenarios.

- Demographic characteristics: Age, gender, years of experience as a PHN, workplace (prefectural, municipal, or core city), pregnancy status, number and age of children, and job title. These items were factual and did not require psychometric validation.
- Knowledge about radiation: This section included several multiple-choice or yes/no items addressing the understanding of radiation concepts (e.g., differences between radiation and radioactivity, internal vs. external exposure, stochastic vs. deterministic effects, dose limits). For instance, one question asked participants to identify the types of radioactive materials detected in tap water after the Fukushima disaster, while another asked about the minimum dose level that causes health effects. Each item was treated individually rather than as part of a composite scale, and correct/incorrect responses were based on established scientific facts. No formal validation (e.g., factor analysis, reliability testing) was conducted on this section, as the items were not intended to form a single latent construct but to gauge general knowledge.
- Awareness of disaster preparedness: Items assessed whether participants knew about the Oita Prefecture nuclear disaster manual, recognized that Oita Prefecture is a designated evacuation site, and knew where evacuees would be received. These questions were factual and contextual, not derived from a standardized scale, and did not undergo reliability testing.
- Anxiety about accepting evacuees: Measured on a 4-point Likert-type scale (“Not anxious at all”, “Not very anxious”, “Somewhat anxious”, “Very anxious”). For the analysis, the responses were dichotomized into “Anxious” (combining “Very anxious” and “Somewhat anxious”) and “Not anxious” (combining “Not very anxious” and “Not anxious at all”). This single-item measure was developed specifically for this study’s exploratory purpose and was not derived from an existing validated tool.
- Interest in training on nuclear disasters: Participants indicated their willingness to participate in training and their preferred formats (online, in-person, etc.). These items were used descriptively and were not psychometrically validated.

Since the questionnaire was exploratory and context-specific, no formal psychometric evaluation (such as Cronbach’s alpha or factor analysis) was performed. The primary aim was to capture a broad range of factors potentially associated with PHNs’ anxiety rather than to measure a single, validated construct.

### 2.5. Statistical Analysis

Descriptive statistics were calculated for all the variables. The univariate analysis used chi-squared tests for categorical variables and Mann–Whitney U tests for continuous variables. The choice of the Mann–Whitney U test for continuous variables was made because the preliminary inspection suggested that the normality assumption may not be met. This non-parametric approach reduces the risk of type I errors when distributions are not normal, although it may lower the statistical power slightly. To explore the generalizability of the findings, we also considered comparing the results using independent *t*-tests, but due to the relatively small sample size (n = 100) and the preliminary evidence of a non-normal distribution, the Mann–Whitney U test was judged to be more appropriate. Nonetheless, should future studies with larger samples confirm the normality, using a parametric test (e.g., *t*-test) could be considered to enhance the generalizability. Variables with a *p*-value <0.2 in the univariate analysis were subsequently entered into a multivariate logistic regression model to identify independent predictors of anxiety. This relatively lenient threshold is commonly used in exploratory research to ensure that potentially important variables are not missed. By allowing variables with a slightly higher *p*-value into the model, we aimed to capture subtle associations that might otherwise be overlooked, thereby providing a more comprehensive evaluation of the potential predictors. Odds ratios (ORs) with 95% confidence intervals (CIs) were calculated, and statistical significance was set at *p* < 0.05. All the variance inflation factors (VIF) in the multivariate analysis were ≤1.255, indicating no multicollinearity concerns (values <2.5 are considered acceptable). The analyses used R software version 4.3.1.

## 3. Results

### 3.1. Participant Characteristics

In Table 1, radiation/disaster-related knowledge and attitudes are presented in the top section, while demographic characteristics are listed in the bottom section. While data on pregnancy, children, and participant roles are included, these showed no significant association with anxiety and are thus provided only for descriptive completeness. Because only two participants were male and only two female participants were pregnant, these factors were not central to the main analysis. The distinction between prefectural, core city, and municipal offices may influence roles and responsibilities during disaster response, and these administrative levels are briefly explained in the table’s footnotes.

A total of 100 public health nurses (PHNs) participated in this study. This clear separation of sections in Table 1 helps readers distinguish demographic characteristics (e.g., age, gender, years of experience, workplace) from survey-based responses (e.g., radiation knowledge, preparedness, and training preferences). The participants’ demographic characteristics are summarized below.

- **Age:** Overall, 23 participants were in their 20s (23.0%), 28 in their 30s (28.0%), 15 in their 40s (15.0%), 33 in their 50s (33.0%), and 1 participant was in their 60s (1.0%).
- **Gender:** Most participants were female (98%).
- **Years of service:** Participants had an average of 16.6 years of experience (SD = 12.1) as PHNs. This value represents the mean and standard deviation (mean [SD]).
- **Workplace:** Participants worked in both prefectural health offices and municipal health centers, with slightly more from municipal settings (52%).

In addition to demographic characteristics, the top section of Table 1 shows participants’ radiation-related knowledge, awareness of disaster preparedness, and training format preferences. For instance, 40.5% of participants preferred online training formats. Although these items are not demographic variables, displaying them together with demographic data provides a comprehensive overview of the sample. Readers should note that these responses represent perceptions and self-reported knowledge rather than fixed participant attributes.

### 3.2. Univariate Analysis

A univariate analysis was used to examine the association between PHNs’ anxiety about accepting evacuees and various factors. The significant findings are summarized below (Table 2).

- **Anxiety about living in Ikata City**: Although not statistically significant, PHNs who felt anxious about living in Ikata City tended to be more anxious about accepting evacuees (*p* = 0.066).
- **Knowledge of evacuation responsibilities:** Although not statistically significant (*p* = 0.11), PHNs who knew of their area’s responsibilities for receiving evacuees showed a lower level of anxiety.
- **Workplace:** Significant differences were observed by workplace. PHNs working in prefectural offices were less anxious than those in municipal offices (*p* = 0.006).

### 3.3. Multivariate Logistic Regression Analysis

A multivariate logistic regression analysis was used to identify independent variables associated with anxiety about accepting evacuees. The results are summarized below (Table 3).

- **Workplace:** PHNs working in prefectural offices were 3.4 times more likely to feel anxious about accepting evacuees than those in municipal offices (OR = 3.488, 95% CI = 1.236–10.963, *p* = 0.023). By contrast, PHNs in core city offices were 2.7 times less likely to feel anxious than those in municipal offices (OR = 0.368, 95% CI = 0.049–1.903, *p* = 0.263), although this result was not statistically significant.
- **Knowledge about evacuation responsibilities:** PHNs who reported knowing of their area’s responsibilities for receiving evacuees were 2.4 times less likely to feel anxious than those who did not know (OR = 0.412, 95% CI = 0.134–1.211, *p* = 0.110), although this result was also not statistically significant.
- **Other variables:** Factors such as anxiety about treated water (*p* = 0.501), anxiety about living in Ikata City (*p* = 0.417), and willingness to receive training via handouts (*p* = 0.280) were not significant independent predictors.

The analysis suggests that workplace characteristics play a crucial role in determining PHNs’ anxiety levels, with those in prefectural offices showing lower levels of anxiety.

## 4. Discussion

### 4.1. PHNs’ Knowledge of Radiation

Although this study’s questions assessed specific factual knowledge about radiation (e.g., differences between stochastic and deterministic effects), the findings primarily reflect certain gaps in PHNs’ understanding rather than a validated measure of their overall radiation knowledge. For example, only 8% correctly identified stochastic vs. deterministic effects, and just 16% knew the general population dose limit, suggesting that some key concepts remain unclear. While these results align with previous studies showing that health professionals often lack sufficient radiation-related understanding [2,6,11], it is important to note that we did not use a standardized, validated test of radiation knowledge. Thus, our findings highlight areas where knowledge may be limited, potentially hindering PHNs’ ability to communicate risks effectively and manage nuclear emergencies, rather than providing a comprehensive assessment of their actual competence.

This could hinder their ability to communicate risks effectively and manage nuclear emergencies.

The low knowledge levels observed in this study highlight the urgent need for targeted educational programs. Studies have shown that well-designed training programs can significantly improve knowledge and confidence in terms of disaster response [12]. Incorporating these educational efforts into the ongoing professional development of PHNs is therefore crucial.

### 4.2. Workplace Differences and Anxiety

The multivariate analysis showed that PHNs working in prefectural offices were 3.4 times more likely to experience anxiety about accepting evacuees than those in municipal offices. In Japan’s administrative framework, prefectural offices oversee broader regional health strategies, municipal offices handle local-level tasks, and core city offices operate at an intermediate administrative level. This structural difference may influence the scope of responsibilities and thus impact anxiety levels. Conversely, those working in core city offices were 2.7 times less likely to feel anxious than municipal office workers. This finding is consistent with previous studies, which suggest that the organizational structure and the scope of responsibilities heavily influence disaster-related stress [7,13].

Prefectural PHNs often play a broader coordination role in large-scale disasters, which can heighten their sense of responsibility and contribute to anxiety [3]. By contrast, core city PHNs may have access to better training resources and clearer operational guidelines, reducing their stress levels [8]. Enhancing communication and support between prefectural and municipal PHNs could help balance these disparities and alleviate anxiety.

### 4.3. Knowledge of Evacuation Sites and Anxiety

Although this difference did not reach statistical significance (*p* = 0.11), PHNs who were aware of evacuation sites tended to be less likely to experience anxiety than those who were unaware. This observation is generally in line with previous disaster preparedness research, which suggests that clear operational knowledge can reduce psychological stress during emergencies [14].

Providing detailed and easily accessible information about evacuation plans and responsibilities is critical. This could be achieved through regular drills, interactive training sessions, and digital resources tailored to specific regional needs. Knowledge dissemination empowers PHNs and enhances their ability to perform effectively during emergencies [5].

### 4.4. Preference for Online Training

Online training was the preferred format among PHNs. This preference reflects the practicality and accessibility of online learning, particularly for busy professionals [15]. Previous studies have highlighted the effectiveness of online platforms in delivering disaster-related education, noting their ability to reach large audiences while maintaining flexibility in terms of time and location [16,17]. Adopting online training programs focused on radiation knowledge and disaster response could therefore address the identified knowledge gaps and cater to PHNs’ preferences. Incorporating hands-on components, such as virtual simulations, could further enhance the learning experience [18,19].

### 4.5. Implications and Recommendations

This study distinguished between demographic characteristics and professional factors (such as radiation knowledge and training preferences). While demographic variables did not strongly influence anxiety, knowledge gaps and workplace roles emerged as more critical. This aligns with the notion that specific professional competencies and contextual responsibilities, rather than personal demographics, shape PHNs’ preparedness and psychological responses in nuclear disaster scenarios.

Our findings suggest several actionable steps for improving PHNs’ preparedness for nuclear disasters:

- Enhanced training programs: Focus on foundational knowledge, such as stochastic versus deterministic effects, and practical skills for managing radiation risks.
- Regional information sharing: Facilitate regular communication between prefectural and municipal offices to harmonize roles and responsibilities.
- Accessible training formats: Leverage online platforms to provide flexible, high-impact education tailored to PHNs’ needs.

### 4.6. Study Limitations

This study is limited by its cross-sectional design, which precludes causal inferences. The response rate of 19.5% may also introduce selection bias, potentially limiting the generalizability of the findings. Furthermore, the questionnaire used in this study was not formally validated, which may limit the reliability and validity of the findings. Future research should aim to validate these results with a larger and more diverse sample. Furthermore, this study’s sample was predominantly female (98%), reflecting the typical gender distribution in the PHN workforce in Japan. While this limits the generalizability of the findings to a more gender-balanced context, it accurately represents the actual PHN demographic in the region studied.

Although demographic factors such as pregnancy status or the presence and age of children were collected, they did not show a clear association with anxiety levels in this study. These factors were included to explore a broad range of potential influences. While not significant here, future research with larger samples could re-examine whether personal life circumstances affect PHNs’ disaster-related anxiety or capacity for response.

Specifically, the questionnaire developed for this study, while grounded in prior research, did not undergo rigorous psychometric validation. This may limit the interpretability of some findings. For instance, in the “Knowledge about radiation” section, only 8% correctly differentiated between stochastic and deterministic effects, and 16% knew the general population dose limit. These items highlight potential knowledge gaps rather than providing a definitive assessment of radiation knowledge. Future studies should utilize validated scales to measure radiation knowledge comprehensively.

Furthermore, future research should aim to validate these results with a larger and more diverse sample and investigate the long-term impact of tailored training programs on PHNs’ preparedness and psychological resilience.

## 5. Conclusions

This study identified key factors that influence PHNs’ anxiety about accepting evacuees during a nuclear disaster. There were significant gaps in the PHNs’ knowledge of radiation and this study highlighted the role of workplace characteristics and awareness of evacuation site responsibilities in shaping anxiety levels. PHNs working in prefectural offices experienced higher anxiety, perhaps due to their broader responsibilities. However, PHNs with knowledge of evacuation sites were less likely to be anxious.

To address these challenges, it may be helpful to introduce targeted interventions, such as comprehensive training programs focused on radiation knowledge and disaster preparedness, and enhanced communication and collaboration across organizational levels. Online training emerged as a preferred and effective format for addressing PHNs’ educational needs.

Future research should validate these findings in larger and more diverse populations, while also exploring the long-term impact of tailored training programs on PHNs’ preparedness and psychological resilience. Strengthening PHNs’ knowledge and confidence is critical to ensuring effective disaster response and supporting evacuees during nuclear emergencies.

## Figures and Tables

**Table 1:** Descriptive statistics concerning the PHNs working in Oita Prefecture (n = 100).

Question Items	Items	n (%)
[Radiation/Disaster Related Responses]		
Do you feel anxious about any of these issues?		
X-rays	Yes	6 (6.0)
	Somewhat yes	14 (14.0)
	Somewhat no	42 (42.0)
	No	38 (38.0)
Foods from Fukushima	Yes	2 (2.0)
	Somewhat yes	11 (11.0)
	Somewhat no	37 (37.0)
	No	50 (50.0)
Going to Fukushima	Yes	1 (1.0)
	Somewhat yes	13 (13.0)
	Somewhat no	33 (33.0)
	No	53 (53.0)
Treated water	Yes	12 (12.0)
	Somewhat yes	43 (43.0)
	Somewhat no	30 (30.0)
	No	15 (15.0)
Living in Ikata City	Yes	10 (10.0)
	Somewhat yes	48 (48.0)
	Somewhat no	28 (28.0)
	No	14 (14.0)
Level of knowledge		
Differences between radiation and radioactive	Known	45 (45.0)
	Unknown	55 (55.0)
The three principles of external radiation exposure	Known	33 (33.0)
	Unknown	67 (67.0)
Differences between internal and external radiation exposure	Known	51 (51.0)
	Unknown	49 (49.0)
Differences between stochastic and deterministic effects	Known	8 (8.0)
	Unknown	92 (92.0)
The limit dose among general population during peacetime	Correct	16 (16.0)
	Incorrect	84 (84.0)
Minimum dose level that causes health effects	Correct	12 (12.0)
	Incorrect	88 (88.0)
The kinds of radioactive materials detected in tap water after the Fukushima disaster	Correct	20 (20.0)
	Incorrect	80 (80.0)
Views on knowledge and training		
Do you think you have enough knowledge about radiation and nuclear disasters?	Yes	0 (0.0)
	Somewhat yes	4 (4.0)
	Somewhat no	44 (44.0)
	No	52 (52.0)
Do you think knowledge of radiation is necessary for public health nursing activity?	Yes	10 (10.0)
	Somewhat yes	81 (81.0)
	Somewhat no	7 (7.0)
	No	2 (2.0)
Do you think knowledge of radiation is necessary among residents?	Yes	10 (10.0)
	Somewhat yes	81 (81.0)
	Somewhat no	7 (7.0)
	No	2 (2.0)
Do you think delayed effects of radiation, such as cancer, will occur among people currently living in Fukushima Prefecture?	Strongly agree	2 (2.0)
	Agree	57 (57.0)
	Disagree	40 (40.0)
	Strongly disagree	1 (1.0)
Do you think hereditary effects of radiation exposure will occur among the children or grandchildren of people currently living in Fukushima Prefecture?	Strongly agree	1 (1.0)
	Agree	32 (32.0)
	Disagree	62 (62.0)
	Strongly disagree	5 (5.0)
Are you aware of the nuclear disaster manuals created by Oita Prefecture?	Known	18 (18.0)
	Heard	29 (29.0)
	Unknown	53(53.0)
Did you know that Oita Prefecture is a designated recipient of evacuees in the event of a nuclear disaster at the Ikata Nuclear Power Plant?	Known	63 (63.0)
	Unknown	37 (37.0)
Do you know where to receive evacuees in your area of responsibility in the event of a nuclear disaster at the Ikata Nuclear Power Plant?	Known	10 (10.0)
	Heard	15 (15.0)
	Unknown	75 (75.0)
Do you have any concerns about accepting evacuees in the event of a nuclear disaster at the Ikata Nuclear Power Plant?	Anxious	4 (4.0)
	Somewhat anxious	38 (38.0)
	Mostly not worried	38 (38.0)
	Not worried at all	20 (20.0)
Have you ever participated in a workshop about nuclear disaster?	Yes	13 (13.0)
	No	87 (87.0)
Have you ever participated in training about nuclear disasters?	Yes	11 (11.0)
	No	89 (89.0)
Would you be willing to participate in a training course on radiation exposure and nuclear disaster prevention if such a course was offered?	Yes	22 (22.0)
	Somewhat yes	64 (64.0)
	Somewhat no	12 (12.0)
	No	2 (2.0)
If training on nuclear disaster prevention was provided in the future, what kind would you prefer? (Multiple answers allowed)	Face-to-face training	26 (26.0)
	Web-based training	85 (85.0)
	Distribution of materials	37 (37.0)
	Hands-on training	26 (26.0)
What style of training would you prefer to receive (not only on nuclear disaster prevention)? (Multiple answers allowed)	Face-to-face training	24 (24.0)
	Web-based training	88 (88.0)
	Distribution of materials	36 (36.0)
	Hands-on training	21 (21.0)
[Demographic information]		
Age	20s	23 (23.0)
	30s	28 (28.0)
	40s	15 (15.0)
	50s	33 (33.0)
	60s or older	1 (1.0)
Gender	Male	2 (2.0)
	Female	98 (98.0)
Pregnancy *^1^	Yes	2 (2.0)
	No	98 (98.0)
Children	None	37 (37.0)
	Less than 1 year old	1 (1.0)
	1–6 years old	14 (14.0)
	7–12 years old	16 (16.0)
	13–18 years old	8 (8.0)
	19 years old or more	24 (24.0)
Workplace *^2^	Prefectural office	32 (32.0)
	Core city office	8 (8.0)
	Municipality office	60 (60.0)
Position	Staff	51 (51.0)
	Unit chief	19 (19.0)
	Assistant section chief	22 (22.0)
	Section chief	7 (7.0)
	Deputy director	1 (1.0)
	Director	0 (0.0)
Year of experience as a public health nurse: Mean (SD)		16.6 (12.1)

Note. Above are radiation/disaster-related response items and below are demographic characteristics. *^1^: The 2 male participants were not asked this question. *^2^: Prefectural office, administrative health offices managed at the prefectural level; core city office, health centers in core cities with certain administrative functions; municipal office, local city/town/village health centers. These distinctions are specific to Japan’s administrative structure.

**Table 2:** Univariate analysis of the associations with PHNs’ anxiety about accepting evacuees.

Question Items	Items	Anxiety About Acceptance		*p*-Value
		Yes (n = 58)	No (n = 42)	
Are you at all anxious about any of these issues?				
X-rays	Yes	10 (17.2)	10 (23.8)	0.455
Foods from Fukushima	Yes	5 (8.6)	8 (19.0)	0.143
Going to Fukushima	Yes	7 (12.1)	7 (16.7)	0.567
Treated water	Yes	30 (51.7)	28 (66.7)	0.155
Living in Ikata City	Yes	27 (46.6)	28 (66.7)	0.066
Knowledge				
Differences between radiation and radioactive	Known	29 (50.0)	16 (38.1)	0.309
The three principles of external radiation exposure	Known	21 (36.2)	12 (28.6)	0.519
Differences between internal and external radiation exposure	Known	31 (53.4)	20 (47.6)	0.686
Differences between stochastic and deterministic effects	Known	4 (6.9)	4 (9.5)	0.717
The limit dose among general population during peacetime	Correct	11 (19.0)	5 (11.9)	0.415
Minimum dose level that causes health effects	Correct	9 (15.5)	3 (7.1)	0.232
The kinds of radioactive materials detected in tap water after the Fukushima disaster	Correct	10 (17.2)	10 (23.8)	0.455
Views on training and knowledge				
Do you think you have enough knowledge about radiation and nuclear disasters?	Yes	1 (1.7)	3 (7.1)	0.307
Do you think knowledge of radiation is necessary for public health nursing activity?	Yes	56 (96.6)	40 (95.2)	>0.999
Do you think knowledge of radiation is necessary among residents?	Yes	52 (89.7)	39 (92.9)	0.730
Do you think delayed effects of radiation, such as cancer, will occur among people currently living in Fukushima Prefecture?	Agree	34 (58.6)	25 (59.5)	>0.999
Do you think hereditary effects of radiation exposure will occur among the children or grandchildren of people currently living in Fukushima Prefecture?	Agree	15 (25.9)	18 (42.9)	0.088
Are you aware of the nuclear disaster manuals created by Oita Prefecture?	Known	10 (17.2)	8 (19.0)	>0.999
Did you know that Oita Prefecture is a designated recipient of evacuees in the event of a nuclear disaster at the Ikata Nuclear Power Plant?	Known	37 (63.8)	26 (61.9)	>0.999
Do you know where to receive evacuees in your area of responsibility in the event of a nuclear disaster at the Ikata Nuclear Power Plant?	Known	11 (19.0)	14 (33.3)	0.110
Have you ever participated in a workshop about nuclear disasters?	Yes	7 (12.1)	6 (14.3)	0.771
Have you ever participated in training about nuclear disasters?	Yes	6 (10.3)	5 (11.9)	>0.999
Would you be willing to participate in a training course on radiation exposure and nuclear disaster prevention if such a course was offered?	Yes	48 (82.8)	38 (90.5)	0.384
If training on nuclear disaster prevention was provided in the future, what kind would you prefer? (Multiple answers allowed)	Face-to-face	12 (20.7)	12 (28.6)	0.477
	Web-based	17 (29.3)	19 (45.2)	0.139
	Distribution of materials	50 (86.2)	38 (90.5)	0.757
	Hands-on	10 (17.2)	11 (26.2)	0.325
What style of training would you prefer to receive? (Multiple answers allowed)	Face-to-face	13 (22.4)	13 (31.0)	0.364
	Web-based	20 (34.5)	17 (40.5)	0.675
	Distribution of materials	49 (84.5)	36 (85.7)	>0.999
	Hands-on	13 (22.4)	13 (31.0)	0.364
Demographic information				
Age	20s	16 (27.6)	7 (16.7)	0.636
	30s	15 (25.9)	13 (31.0)	
	40s	9 (15.5)	6 (14.3)	
	50s	17 (29.3)	16 (38.1)	
	60s or older	1 (1.7)	0 (0.0)	
Gender	Female	57 (98.3)	41 (97.6)	>0.999
Pregnancy	Yes	2(3.4)	0 (0.0)	0.508
Children	None	24 (41.4)	13 (31.0)	0.500
	Less than 1 year old	0 (0.0)	1 (2.4)	
	1–6 years old	10 (17.2)	4 (9.5)	
	7–12 years old	8 (13.8)	8 (19.0)	
	13–18 years old	4 (6.9)	4 (9.5)	
	19 years old or more	12 (20.7)	12 (28.6)	
Workplace	Prefectural office	25 (43.1)	7 (16.7)	0.006
	Core city office	2 (3.4)	6 (14.3)	
	Municipality office	31 (53.4)	29 (69.0)	
Position	Staff	16 (27.6)	14 (33.3)	0.659
	Unit chief or higher	42 (72.4)	28 (66.7)	
Years of experience as a public health nurse		15.7 (12.3)	17.9 (11.8)	0.352

**Table 3:** Multivariate logistic regression analysis of the associations with PHNs’ anxiety about accepting evacuees (n = 100).

Questions	Items	Estimate	OR	95% CI		*p*-Value	VIF
				Lower	Upper		
Do you feel anxious about treated water?	No (reference)						
	Yes	−0.335	0.716	0.268	1.909	0.501	1.222
Do you feel anxious about living in Ikata City?	No (reference)						
	Yes	0.400	1.492	0.567	3.969	0.417	1.186
Do you think hereditary effects of radiation exposure will occur among the children or grandchildren of people currently living in Fukushima Prefecture?	No (reference)						
	Yes	−0.161	0.851	0.307	2.392	0.757	1.255
Do you know where to receive evacuees in your area of responsibility in the event of a nuclear disaster at the Ikata Nuclear Power Plant?	No (reference)						
	Yes	−0.887	0.412	0.134	1.211	0.110	1.169
Would you like to receive training in the style of a handout?	No (reference)						
	Yes	0.505	1.657	0.661	4.178	0.280	1.041
Workplace	Municipal office (reference)						
	Core city office	−0.998	0.368	0.049	1.903	0.263	1.156
	Prefectural office	1.249	3.488	1.236	10.963	0.023	1.156

## Data Availability

All the data are available from the corresponding author upon reasonable request.
